# University Students’ Willingness to Assist Fellow Students Who Experience Alcohol-Related Facial Flushing to Reduce Their Drinking

**DOI:** 10.3390/ijerph15050850

**Published:** 2018-04-25

**Authors:** Lanyan Ding, Lok-Wa Yuen, Ian M. Newman, Duane F. Shell

**Affiliations:** Department of Educational Psychology, University of Nebraska, Lincoln, NE 68588-0345, USA; lok-wa.yuen@huskers.unl.edu (L.-W.Y.); inewman1@unl.edu (I.M.N.); dshell2@unl.edu (D.F.S.)

**Keywords:** ALDH, China, alcohol, ethanol, aerodigestive cancer, esophageal cancer, bystander, education

## Abstract

This study explored bystanders’ willingness to help a friend who flushes when drinking to reduce his/her drinking. Alcohol-related facial flushing is an indicator of an inherited variant enzyme, aldehyde dehydrogenase (ALDH), that impairs alcohol metabolism and increases drinkers’ lifetime risk of certain aerodigestive cancers. Individuals who flush should reduce their alcohol exposure, but they may continue to drink if social pressures and rules of etiquette make not drinking socially risky. The analysis used data from 2912 undergraduate students from 13 universities in southwestern, central and northeastern China from a survey asking how they respond to someone’s flushing in various scenarios. Latent class analysis grouped students by similar responses to flushing. A multinomial logistic regression explored how class membership was associated with knowledge, drinking status, and reactions to one’s own flushing. Five classes were derived from the latent class analysis, ranging from *always intervene* to *mostly hesitate* to help; in between were classes of students who were willing to help in some scenarios and hesitant in other scenarios. Only 11.6% students knew the connection between facial flushing and impaired alcohol metabolism, and knowledgeable students were somewhat more likely to assist when they saw someone flushing. In the absence of knowledge, other factors—such as drinking status, the gender of the bystander, the gender of the person who flushed, and degree of friendship with the person who flushed—determined how willing a person was to help someone reduce or stop drinking. Class membership was predicted by knowledge, gender, drinking status, and reactions to one’s own flushing. Of these 4 factors, knowledge and reactions to one’s own flushing could be influenced through alcohol education programs. It will take some time for alcohol education to catch up to and change social and cultural patterns of drinking. Meanwhile, motivational strategies should be developed to increase the willingness of bystanders to assist friends and to create a social expectation that flushers should stop or reduce their drinking.

## 1. Introduction

Ethanol-induced facial flushing is a physiological reaction to alcohol consumption in which the drinker’s face and neck turns red. The person whose face flushes may also experience other feelings of discomfort, such as an elevated heart rate, headache, heart palpitation, shortness of breath, hyperventilation, low blood pressure, vertigo, nausea, and vomiting [1]. Flushing results from the body’s compromised ability to metabolize alcohol, allowing acetaldehyde to build up, which increases a person’s risk of cancers of the respiratory tract and upper digestive tract, including the lips, mouth, tongue, nose, throat, and vocal cords [2]. Individuals with the ALDH2*1 gene typically flush when drinking, and they tend to have compromised alcohol metabolism with the associated health risks. Flushing is not a complete indicator of ALDH2*1 but it is an excellent proxy. About half of Chinese, Japanese, Korean and other Asian people have this genetic predisposition to flush [2,3]. Despite the associated discomforts and risks, individuals who flush often drink through their discomfort and develop some level of tolerance for alcohol [4]. Possible reasons for drinking despite the discomfort are the lack of knowledge about what causes flushing and the wide spread social pressures to continue drinking.

The World Health Organization (WHO) estimates a 37% increase in adult per capita alcohol consumption of pure alcohol among those aged 15 and above in China between 2008 and 2010 [5]. In traditional Chinese culture, moderate drinking is important in special celebrations, festivals and is considered good for health. Drinking with others is an important part of establishing and maintaining social relationships. A recent meta-analysis of drinking among university students indicated a last-30-day drinking rate of 66.8% for males and 31.7% for females [6].

Flushing is a clearly visible sign of compromised alcohol metabolism; therefore, fellow drinkers could be expected to notice someone flushing and encourage that person to slow down or stop drinking. However, surveys of Chinese university students revealed considerable lack of knowledge of the significance of flushing as well as a reluctance to discourage flushing drinkers from further drinking. Of three surveys of Chinese university students from different parts of the country [7,8,9], in only one did a majority of students (50.4%) say they would suggest that flushing students stop or reduce their drinking. Students in the more knowledgeable sample were enrolled for education and medical degree programs whereas students in the other two samples were enrolled in multiple disciplines. These earlier surveys suggested a need to increase knowledge about why a person flushes and build support for flushers to reduce their alcohol intake [9].

Previous research suggests that gender and drinking status are important in determining who in a group would encourage a flushing drinker to stop or reduce their drinking. Newman et al. [8] reported that females were more likely to encourage a flusher to stop drinking, regardless of the gender of the person flushing. Non-drinkers were more likely to suggest a flusher stop or reduce drinking. There is also evidence to suggest that responses to a flushing drinker are related to the degree of friendship. One recent study suggested the likelihood peers will encourage a flushing drinker to reduce or stop drinking was related to the degree of friendship with the flusher and purpose of the drinking occasion [10]. Students were less likely to intervene with a flushing drinker if they did not know the flusher, but more likely to intervene if the flusher was a close friend. However, friendship may not always influence behavior, especially in China where there are many rules about relationships and etiquette within drinking groups. The purpose of the drinking occasion also seems to affect the likelihood a fellow student will assist. People were more likely to help a flushing drinker reduce or stop drinking when in a relaxed drinking environment where the purpose is to enjoy socializing. In contrast, when drinking in an environment where heavy drinking, often for celebratory purposes, is occurring, any concern for the flushing drinker is overridden by etiquette requirements and drinking goals [10,11].

This limited number of studies has suggested that future attempts to encourage flushers and bystanders to modify their behaviors will be complex and influenced by social and environmental factors. For this study, we used latent class analysis (LCA) to identify and describe different groups of students based on their willingness to intervene and encourage a fellow drinker who is flushing to reduce or stop their drinking. Then we assessed the association between class membership and covariates to see what variables predicted class membership. Last, we discuss which of the significant covariates could be influenced in order to create more willingness to help someone who is flushing. LCA has been used to study drinking settings [12], severity of alcohol dependence [13], typology of disorders [14,15,16,17], but has not previously been used to describe reactions to alcohol-related facial flushing.

## 2. Materials and Methods

### 2.1. Subjects

A total of 2912 undergraduate students from 13 universities in southwestern, central and northeastern China completed a survey which collected data about alcohol use, alcohol-related flushing, and demographic characteristics. Surveys were given by experienced data collectors in classes selected at random and all students in the selected classes were asked to complete the survey. The survey was anonymous, and students had the option to not complete the survey.

### 2.2. Measures

*Reactions to a flusher.* Respondents were asked to report their response when someone flushed while drinking in different types of drinking scenarios. The eight drinking scenarios presented variations in the gender of flusher, degree of friendship, and risk level of drinking situation: (1) a male close friend in a fun drinking situation; (2) a male close friend in a risky drinking situation; (3) a male general friend in a fun drinking situation; (4) a male general friend in a risky drinking situation; (5) a female close friend in a fun drinking situation; (6) a female close friend in a risky drinking situation; (7) a female general friend in a fun drinking situation; and (8) a female general friend in a risky drinking situation. The survey provided three options for each scenario: (1) I would encourage the flusher to reduce or stop drinking; (2) I would not encourage the flusher to reduce or stop drinking even though I feel that I should; and (3) I would not do anything because there is no need to. Examples of drinking scenarios are included in Appendix A.

*Knowledge of flushing.* Students’ knowledge of alcohol-related flushing was assessed by a question that asked about the reason for flushing “What does it mean if a person flushes when drinking alcohol?” Five response options were provided: (1) flusher is good at drinking; (2) flusher’s body can get rid of alcohol more efficiently; (3) flusher is sensitive to the alcohol; (4) flusher’s body cannot metabolize the alcohol completely; and (5) some other meaning. Respondents were allowed to choose more than one answer though only one of the four choices was correct. Respondents were dichotomized. Respondents who solely selected the correct answer (option 4) were classified as having correct knowledge of flushing; all others, including those who selected both the correct answer and one or more incorrect answers, were classified as having incorrect knowledge about flushing. For analysis, knowledge about flushing was converted into a dummy variable (incorrect knowledge = 0, correct knowledge = 1).

*Reactions to one’s own flushing.* Students’ reactions to their own flushing was measured by asking students what they did when they flushed while drinking. The three response options were: (1) I never drink or flush; (2) I stop drinking; and (3) I continue drinking. Reaction to one’s own flushing was converted into two dummy variables (ACT1 = do not drink or flush, ACT2 = continue drinking).

*Drinking status.* Drinking status was based on self-reported frequency of drinking in the past 30 days. Students who reported never drinking or not drinking in the past 30 days were classified as nondrinkers, and students who reported drinking alcohol within the last 30 days were classified as drinkers. Drinking status was dummy coded (nondrinker = 0, drinker = 1).

*Gender.* Gender was dummy coded (female = 0, male = 1).

### 2.3. Data Analysis

We used the latent class analysis (LCA) to identify heterogeneous classes of individuals with shared similar characteristics in response patterns and then examined selected covariates to identify predictors of class membership.

A 3-step procedure of LCA (identification of latent class, creation of the most likely class variable, and regression of the most likely class on covariates) was conducted using Mplus 7.0 [18]. We used LCA to identify the number and nature of sub-types of willingness to intervene based on the 8 scenarios, each with 3 responses, resulting in 24 item responses. We began with a three-class model, then added one additional class at a time until a six-class model evolved. We chose the number of classes according to: (1) best fit structure of the data (i.e., goodness-of-fit indices); and (2) classes that represented theoretically and practically meaningful patterns [19,20,21,22]. Specifically, the statistical fit indices were the loglikelihood value, Akaike information criterion (AIC) [23], Bayesian information criterion (BIC) [24], sample-size adjusted BIC (SSABIC) [25], the Lo-Mendell-Rubin’s adjusted likelihood ratio test (aLRT) [26], and entropy measures [27].

To interpret the item-response probabilities of the responses to a person who is flushing (suggest, do not suggest but feel I should, and do nothing) we chose to focus on the highest probabilities for each scenario. Since there were eight scenarios the responses with at least two of the three highest probabilities would be seen as representing the latent classes. If a reaction to a flusher had only two highest probabilities and any of the two were lower than 0.4, then we judged these reactions to a flushing student to be uncertain. The highest probabilities are shaded in gray in the table in Appendix B.

Following the identification of class membership, all covariates were entered simultaneously into a multinomial logistic regression predicting class membership to explore how classes might be differentially associated with the variables. The class that included individuals with the highest probability of willingness to intervene to help a flusher in reducing drinking was used as the reference group.

### 2.4. Ethical Approval

The survey data upon which the results are based are available at Newman et al. [28]. Responsible authorities at each university approved the project and the surveys. The University of Nebraska-Lincoln Institutional Research Board approved both the qualitative and quantitative procedures prior to the study (survey development: IRB #20100610932EX; survey: IRB #20110500663EP). 

## 3. Results

Data from 2561 (87.9%) of the surveys presented valid responses (44.6% male and 55.4% female; 0.4% under age 18, 90.4% aged 18–22, 8.5% over age 22, and 0.7% did not report age; 53.7% first-year students, 17.1% second-year, 17.3% third-year, 9.2% fourth-year students, and 2.6% not reporting year).

Of the 2561 Chinese undergraduate university students presenting valid responses, 296 (11.6%) correctly answered a question about what causes flushing: the flusher’s body cannot metabolize the alcohol completely. Incorrect answers were: the flusher is sensitive to alcohol 1619 (63.2%), the flusher’s body can get rid of alcohol more efficiently 645 (25.2%), and the flusher is good at drinking 272 (10.6%). Regarding a student’s reaction to his/her own flushing: 400 (15.6%) stopped drinking when they flushed, 1107 (43.2%) continued drinking, 977 (38.1%) students reported no flushing/drinking experience, and 77 (3.0%) did not report their reaction to own flushing. Last-30-day alcohol use for this sample was: 1018 (39.8%) students drank in the last 30 days, 1523 (59.5%) students did not drink in the last 30 days, and 20 (0.8%) did not report their drinking behavior.

### 3.1. Classes of Response to Flushing

The fit of four models (three-class to six-class model) was assessed using LCA, and the fit indices are reported in Table 1. From a statistical point of view, smaller AIC and BIC indicate better model fit. Although the six-class solution had the smallest AIC and BIC, the model fit did not improve significantly compared to a five-class solution (aLRT = 1541.45, *p* = 0.1389). From a practical point of view, the five-class solution formed more interpretive classes than other solutions. Therefore, five classes were extracted. Details for the conditional item-response probabilities within each class membership are presented in Appendix B.

Class 1 (13.6% of the population) was labeled *mostly hesitate* class. Students in this class tended to hesitate to intervene when the flusher is a close friend or a general friend regardless of gender. The probabilities of being in the *mostly hesitate* class were 0.50 to 0.85. Yet, when a female close friend flushes, they reported some willingness to intervene.

Class 2 (31.6% of the population) was labeled *do nothing in risky situations* class. In a risky drinking occasion (celebratory, heavy, to get drunk), students in this class were likely to do nothing when the flusher is a male regardless of friendship and to hesitate to intervene when a female general friend flushes, but they were willing to stop a female close friend. In the opposite situation of drinking for fun, they reported willingness to intervene when the flusher is a female or a male close friend, but they would do nothing for a male general friend.

Class 3 (11.6% of the population) was labeled *intervene for close friends* class. Students in this class were willing to intervene for close friends but hesitate to intervene for general friends, regardless of drinking purposes and gender of the flusher. The probabilities of intervening with a close friend ranged between 0.74 and 1.00, and the probabilities of hesitating to intervene on behalf of a general friend ranged between 0.81 and 0.94.

Class 4 (32.9% of the population) was labeled *intervene for females* class. Students in this class were willing to intervene on behalf of female flushers regardless of friendship and drinking purposes. The probabilities of intervening ranged between 0.51 and 0.98. Students in this class were usually hesitant to intervene for a male general friend when he flushes, with the probabilities of being hesitant ranged between 0.51 and 0.64.

Class 5 (10.4% of the population) was labeled *always intervene* class. Students in this class were the most likely to intervene, regardless of friendship, drinking purposes, and gender of the flusher. When they see someone flush, they would always encourage the person to stop drinking or to drink less. The probabilities of intervening on behalf of a flusher were 0.88 to 1.00.

### 3.2. Factors Associated With Classes of Response to Flushing

With these five sub-population groups defined, Table 2 presents the frequency and percentages for gender, drinking status, knowledge of flushing, and reaction to own flushing in each latent class. There were significant associations between the latent classes and knowledge of flushing (𝜒^2^ = 11.16, *p* = 0.025), reaction to own flushing (𝜒^2^ = 19.31, *p* = 0.013), drinking status in the last 30 days (𝜒^2^ = 48.52, *p* < 0.001), and gender (𝜒^2^ = 48.80, *p* < 0.001).

Multinomial logistic regression was applied to examine how knowledge of flushing, gender, drinking status, and reactions to one’s own flushing would predict willingness-to-intervene class membership. The ideal behavior is to intervene every time when seeing someone in the drinking group flush, so Class 5 (*always intervene*), those with the highest probability of intervening, was used as a reference group to compare the other classes (Table 3).

The reaction to one’s own flushing influenced the membership in Class 1 (*mostly hesitate*) versus Class 5 (*always intervene*). Compared to Class 5 (*always intervene*), students who had no experience in flushing or drinking had higher odds of being in Class 1 (*mostly hesitate*) than students who stopped drinking when they flushed, holding all other variables constant. Students who continued drinking after they flushed also had higher odds of being in Class 1 (*mostly hesitate*) versus Class 5 (*always intervene*) than students who stopped drinking when they flushed. Knowledge of flushing, drinking status, and gender were not predictive of membership in Class 1 (*mostly hesitate*).

The odds of being in Class 2 (*do nothing in risky situations*) versus Class 5 (*always intervene*) were higher for students who did not have correct knowledge about the cause of flushing, with all other variables holding constant. Students who continued drinking after flushing also had higher odds of being in Class 2 (*do nothing in risky situations*) than students who stopped drinking after flushing, relative to Class 5 (*always intervene*). Students who had no experience in flushing or drinking had similar odds of being in Class 2 (*do nothing in risky situations*) as students who stopped drinking when they flushed. The odds of being in Class 2 (*do nothing in risky situations*) were not different for drinkers or nondrinkers or males and females.

Membership in Class 3 (*intervene for close friends*) versus Class 5 (*always intervene*) was predicted by drinking status and gender. Students who did not drink in the last 30 days had higher odds of being in Class 3 (*intervene for close friends*) than in Class 5 (*always intervene*) than students who drank, holding all other variables constant. Female students also had higher odds of being in Class 3 (*intervene with close friend*) than male students. Knowledge of flushing and reaction to one’s own flushing were not associated with membership in Class 3 (*intervene for close friends*) versus Class 5 (*always intervene*).

Knowledge of flushing, drinking status, gender, and reaction to one’s own flushing did not predict membership in Class 4 (*intervene for females*) versus Class 5 (*always intervene*).

## 4. Discussion

This study explored variables that are associated with the willingness to assist a person who flushes while drinking alcohol to drink less or stop drinking. The flushing is an indication they may have an impaired capacity for metabolizing alcohol, which carries greater risk for certain alcohol-related cancers. In China, social drinking is an expected component of hospitality, business alliances, friendships, the celebration together of life’s joyful events, and the mourning together of life’s sorrowful events. To refuse a drink may be perceived as a breach of etiquette, may cause social embarrassment, and even lead to misunderstandings. Even people who flush when they drink will keep drinking as a courtesy to other people [4,29,30,31,32]. However, because of their physiology, it is preferable for people who flush (as much as 50% of China’s population, by some estimates) to stop drinking when they flush [2,3,4]. Since facial flushing is also apparent to others it would be desirable for others (bystanders) to encourage the person who is flushing to stop drinking. Even though Western literature has described the role of bystanders in alcohol, smoking [33], sexual violence [34,35], and bullying [36] situations, it is unclear exactly how “bystanders” might react in Chinese situation, as evidenced by these results.

The five latent classes derived from the survey data aligned with Chinese social expectations around gender roles, male drinking behavior, friendship obligation, and perceptions of drinking “rules” that vary according to drinking purpose, setting, and companions. The variables that significantly predicted class membership were: (1) knowledge about the cause of flushing; (2) gender of participants; (3) last-30-day drinking status; and (4) a participant’s reaction to their own flushing. The discovery of these variables may allow educators to make inroads into drinking behaviors in China that would reduce alcohol use for individuals who flush when they drink. Knowledge would be the easiest variable to change [9].

This analysis noted that accurate information about the links between facial flushing and impaired alcohol metabolism are not widely known. Only 11.6% of this sample could answer the knowledge question accurately. Flushing was interpreted by a proportion of this sample as a sign the drinker could handle lots of alcohol. This finding is in line with earlier studies [7,8,9]. Even though alcohol-induced facial flushing is related to increased risk for certain cancers, we did not ask a knowledge question specifically about flushing and cancer.

The majority of the participants in this survey would intervene to help a female who flushes. A detailed analysis of the effect of gender on willingness-to-intervene is reported in Newman et al. [10]. The logistic regression estimates yielded no significant differences between Class 4 *intervene for females* and Class 5 *always intervene* on the four significant variables. This might be because something stronger than these variables, such as cultural gender-role expectations, is at work that allows females to be excused from drinking when they flush without appearing discourteous. A recent meta-analysis of alcohol use surveys of Chinese university students estimated last-30-day alcohol use at 66.8% for male undergraduate students and 31.7% for female undergraduate students [6]. However, there is evidence that the ratio of men to women drinkers is shrinking [37] due to changes in gender role expectations and more women entering the work force. This analysis indicated that female close friends who flush are securely protected: students in all of the five classes showed willingness to intervene for female close friends while drinking. However, given the upward trend in women’s drinking, it is time to reinforce this social expectation so that it doesn’t erode if women’s drinking rates continue to rise.

Men who flush lack support to stop drinking. Overall, men with the inherited characteristic of slow alcohol metabolism run higher lifetime health risks compared to women due to higher frequency and quantity of male drinking [37]. The overall per-capita alcohol consumption in China increased from an average of 10.61 L of pure alcohol between 2003 and 2005 to 15.1 L of pure alcohol in 2010 [5,38], with males consuming the most (18.7 L pure alcohol annually adult per capita compared to women’s 7.6 L in 2010) [38]. Given this trend we expect more men with impaired alcohol metabolism are drinking alcohol and drinking greater quantities when they do drink. One-third of this sample (31.6%) clustered in Class 2 *do nothing in risky situations*, which was characterized by ignoring male flushing, especially in risky drinking situations. Also, one-eighth of this sample (13.6%) clustered in Class 1 *mostly hesitate* reporting that they would not intervene to help a man who flushed, *but thought that they should*. This group could be viewed as a bloc of potential protectors who could be recruited to help male flushers to stop drinking. Interesting, from our point of view, both membership in Class 2 compared to Class 5 (*always intervene*) as well as membership in Class 1 compared to Class 5 were predicted by continuing to drink after flushing. The regression estimates indicated people who stop drinking when they flush are more willing to assist male flushers to stop drinking. In this sample, 43.2% of students said they continued to drink after flushing. It may be possible to persuade people who currently continue to drink after they flush to change their behavior. In Hendershot et al.’s experiment with Asian-Americans, the experimental group was provided with their ALDH2 genetic test result and alcohol risk information. By post-test, ALDH-deficient individuals in the experimental group had reduced their last-30-day drinking quantity and frequency [39]. We believe accurate information about the cause of alcohol-related facial flushing would raise alcohol risk awareness in a similar way to providing genetic feedback to drinkers, and is more practical and less costly [9,10]. While raising awareness might change the behavior of flushers, less clear is how education would be able to change the behaviors of the drinking companions who pressure flushers to drink more, especially for males [9].

Many students in this sample reported higher level of willingness to help close friends who flush than general friends who flushed. A detailed analysis of the effect that degree-of-friendship had on willingness-to-intervene is reported in Newman et al. [10]. Gender (being female) and no reported last-30-day alcohol use predicted membership in Class 3 *intervene for close friends*. Neither variable is changeable. Members of Class 1 and 3 indicated they would not intervene when a general friend flushed, *but thought that they should*. According to transtheoretical theory of behavior change [40] which discusses how to design behavior change programs based on people’s readiness for action, it might be possible for this bloc of students to be influenced by a stage-based intervention to expand their protective behavior beyond close friends to general friends. Persuasive messages will have to be specific to the cultural context where drinking together signifies and deepens friendship, and where urging someone to have another drink is a mark of the seriousness of one’s feelings. In this sample a large majority (88.4%) of students did not know that the cause of flushing was compromised alcohol metabolism. It would be interesting to see what effect this information would have on friends’ behavior toward friends who flush. Would accurate information mean that in the future the sign of friendship would be to urge a friend who flushes—even a general friend—to stop drinking?

Risky drinking situations are characterized by higher quantities of alcohol, forced drinking, and getting drunk as a proof of one’s commitment to the group. A detailed analysis of the effect of drinking purpose had on willingness-to-intervene is reported in Newman et al. [10]. Risky drinking situations create the highest risks for flushers while at the same time reducing willingness of others to assist flushers to slow down or stop drinking. This analysis found that answering the knowledge question incorrectly increased the likelihood of doing nothing to help male flushers in risky drinking scenarios, which makes it appear that knowledge could be an important element for behavioral change. However, the students in this sample who answered the knowledge question accurately also showed greater hesitation to help flushers in risky drinking scenarios.

### Limitations

Results from this study need to be considered in light of several limitations. The sample was limited to students from 13 universities in different parts of China. We cannot be certain that our findings would generalize to all undergraduate university students in China. Moreover, our results are based on subjects’ reported intentions to encourage a flushing drinker to reduce or stop their drinking. Intentions may not reflect actual behaviors. The identification of the five classes depended upon 24 hypothetical scenarios for alcohol use, which may not have clearly reflected the reality of all students. The potential for misclassification due to measurement variations need to be kept in mind. Nevertheless these results do add new knowledge of how students react to a flushing drinker. This new knowledge is valuable in considering ways to increase the number of students who will intervene to assist a flushing drinker.

## 5. Conclusions

The variables that significantly predicted willingness to assist flushers in various scenarios were knowledge about the cause of flushing, reactions to one’s own flushing, last-30-day drinking, and gender. Of these variables, the knowledge variable is likely the easiest to change. However, as there is no formal alcohol education in primary and secondary schools or at the University level, it will take some time to change long-standing social and cultural attitudes toward drinking. With sustained education about the cause of flushing and its link with life-time cancer risks it may be possible to make some progress in lowering expectations—both in terms of drinking frequency and quantity—for drinkers who flush. This analysis showed flushers who stop drinking in reaction to their own flushing are more likely, when they are bystanders, to assist other people who flush to stop or slow down. With Chinese women’s drinking rates trending upward, now may be the time to reinforce the current expectations among people to protect female drinkers who flush. How to motivate people to assist males who flush in risky drinking situations will be the most difficult problem to solve. This analysis suggests motivational strategies could be developed to create social expectations for bystanders to assist friends who flush to stop or reduce their drinking. In the absence of any formal alcohol education in schools it is worth exploring how social media may serve as a platform for this type of education.

## Figures and Tables

**Table 1:** Goodness-of-Fit Indices Comparing Class Models of Intentions.

Class	Log-Likelihood	# par.	AIC	BIC	SSABIC	aLRT	*p*-Value	Entropy
**3**	−47,564.27	74	95,420.53	96,274.36	95,810.48	5504.82	<0.001	0.911
**4**	−46,345.29	99	93,080.57	94,220.96	93,601.39	2431.64	<0.001	0.913
**5**	−45,326.24	124	91,140.48	92,567.43	91,792.18	2032.80	<0.001	0.919
**6**	−44,553.51	149	89,693.03	91,406.54	90,475.60	1541.45	0.1389	0.924

Note: #par. = number of free parameters.

**Table 2:** Frequency and percentage distribution of class membership by knowledge, gender, drinking status, and reaction to own flushing (numbers in parentheses are the percentage).

Variables	Latent Class				
	Class 1	Class 2	Class 3	Class 4	Class 5
	*Mostly hesitate*	*Do nothing in risky situations*	*Intervene for close friends*	*Intervene for females*	*Always intervene*
	(n = 348)	(n = 808)	(n = 296)	(n = 842)	(n = 267)
**Knowledge of flushing**					
incorrect	303 (87.1%)	736 (91.1%)	253 (85.5%)	745 (88.5%)	228 (85.4%)
correct	45 (12.9%)	72 (8.9%)	43 (14.5%)	97 (11.5%)	39 (14.6%)
**Reaction to own flushing**					
not flush	147 (42.7%)	293 (37.4%)	110 (38.5%)	322 (39.6%)	105 (40.9%)
stop	40 (11.6%)	111 (14.2%)	59 (20.6%)	140 (17.2%)	50 (19.5%)
continue	157 (45.6%)	379 (48.4%)	117 (40.9%)	352 (43.2%)	102 (39.7%)
**Drinking status in the last 30 days**					
nondrinker	225 (65.6%)	412 (51.6%)	215 (72.6%)	514 (61.3%)	157 (59.2%)
drinker	118 (34.4%)	387 (48.4%)	81 (27.4%)	324 (38.7%)	108 (40.8%)
**Gender**					
male	152 (43.7%)	425 (52.6%)	89 (30.1%)	354 (42.0%)	123 (46.1%)
female	196 (56.3%)	383 (47.4%)	207 (69.9%)	488 (58.0%)	144 (53.9%)

Notes: 20 missing values in drink in the last 30 days and 77 missing cases in reaction to own flushing.

**Table 3:** Latent Class Regression Estimates of Gender, Knowledge, Drinking Status and Reaction to Own Flushing using Class 5 (*always intervene*) as Reference.

Covariates	Latent Class							
	Class 1		Class 2		Class 3		Class 4	
	*Mostly hesitate*		*Do nothing in risky situations*		*Intervene for close friends*		*Intervene for females*	
	Est (S.E.)	OR	Est (S.E.)	OR	Est (E.S.)	OR	Est (S.E.)	OR
Knowledge	−0.02 (0.25)	0.98	−0.55 (0.23)	0.58 *	0.08 (0.25)	1.09	−0.23 (0.22)	0.79
Drinker	−0.29 (0.20)	0.75	0.28 (0.16)	1.32	−0.46 (0.20)	0.63 *	−0.05 (0.16)	0.95
Male	−0.15 (0.19)	0.86	0.12 (0.16)	1.12	−0.69 (0.20)	0.50 **	−0.23 (0.16)	0.80
ACT1	0.63 (0.27)	1.88 *	0.21 (0.22)	1.23	−0.10 (0.25)	0.91	0.08 (0.21)	1.08
ACT2	0.77 (0.28)	2.15 *	0.43 (0.22)	1.54 *	0.13 (0.25)	1.13	0.22 (0.21)	1.24

Notes. * *p* < 0.05 and ** *p* < 0.001. Gender: 0 = female, 1 = male. Drinker: 0 = nondrinker, 1 = drinker. Knowledge: 0 = incorrect, 1 = correct. ACT1 and ACT2 are dummy variables for reaction to own flushing (ACT1 = do not flush or not drink for reaction to own flushing, ACT2 = continue drinking for reaction to own flushing), and stop drinking is the reference category.

## Appendix A. Examples of Drinking Scenarios

Below are two examples of the drinking scenarios used in this study:

For these questions, assume that you went out to have a dinner with several friends during a leisurely weekend. The atmosphere was comfortable, and you talked a lot around the dinner table. During the dinner, you noticed one of your friends flushed when drinking alcohol. (Fun)

For these questions assume that you were going to graduate. You and all your classmates got together to enjoy the last group dinner, eating and drinking together. It was very noisy. Many people were speaking loudly, some of them were even crying. During the dinner, one of your friends flushed. (Risk)

## Appendix B

**Table A1:** Conditional Item-Response Probabilities.

	Class 1			Class 2			Class 3			Class 4			Class 5		
	*Mostly Hesitate*			*Do Nothing in Risky Situations*			*Intervene for Close Friends*			*Intervene for Females*			*Always Intervene*		
	Pr(1)	Pr(2)	Pr(3)	Pr(1)	Pr(2)	Pr(3)	Pr(1)	Pr(2)	Pr(3)	Pr(1)	Pr(2)	Pr(3)	Pr(1)	Pr(2)	Pr(3)
**A male close friend flushes**															
Fun 1	0.55	0.42	0.03	0.45	0.19	0.37	0.93	0.03	0.04	0.80	0.15	0.05	0.97	0.01	0.02
Fun 2	0.46	0.50	0.04	0.44	0.17	0.39	0.92	0.04	0.04	0.78	0.17	0.05	0.97	0.02	0.01
Fun 3	0.21	0.72	0.07	0.41	0.25	0.34	0.93	0.01	0.06	0.72	0.24	0.04	1.00	0.00	0.00
Risky 1	0.21	0.69	0.11	0.23	0.21	0.55	0.75	0.12	0.14	0.50	0.37	0.13	0.97	0.03	0.01
Risky 2	0.32	0.64	0.05	0.40	0.27	0.33	0.95	0.03	0.03	0.72	0.24	0.04	1.00	0.01	0.00
Risky 3	0.15	0.71	0.15	0.19	0.12	0.70	0.74	0.10	0.16	0.38	0.41	0.22	0.99	0.01	0.00
**A female close friend flushes**															
Fun 1	0.76	0.22	0.02	0.75	0.11	0.14	1.00	0.00	0.00	0.98	0.02	0.00	0.99	0.01	0.01
Fun 2	0.66	0.32	0.01	0.70	0.15	0.16	0.96	0.03	0.01	0.96	0.03	0.00	0.99	0.01	0.00
Fun 3	0.41	0.57	0.03	0.62	0.21	0.17	1.00	0.00	0.00	0.91	0.08	0.01	1.00	0.00	0.00
Risky 1	0.49	0.48	0.03	0.50	0.19	0.31	0.90	0.07	0.03	0.82	0.16	0.02	1.00	0.00	0.00
Risky 2	0.54	0.44	0.02	0.62	0.22	0.17	0.99	0.01	0.00	0.91	0.09	0.01	1.00	0.00	0.00
Risky 3	0.34	0.61	0.04	0.32	0.18	0.50	0.91	0.05	0.04	0.65	0.29	0.06	1.00	0.00	0.00
**A male general friend flushes**															
Fun 1	0.12	0.77	0.11	0.18	0.43	0.40	0.06	0.88	0.06	0.38	0.58	0.05	0.88	0.11	0.02
Fun 2	0.21	0.65	0.14	0.28	0.33	0.40	0.08	0.87	0.06	0.51	0.44	0.05	0.95	0.05	0.00
Fun 3	0.04	0.78	0.18	0.22	0.37	0.41	0.04	0.91	0.05	0.43	0.51	0.06	0.98	0.02	0.00
Risky 1	0.08	0.68	0.24	0.12	0.30	0.58	0.01	0.87	0.12	0.22	0.64	0.14	0.94	0.05	0.01
Risky 2	0.06	0.80	0.14	0.17	0.43	0.40	0.01	0.93	0.06	0.35	0.59	0.07	0.97	0.03	0.00
Risky 3	0.05	0.69	0.27	0.11	0.17	0.72	0.04	0.81	0.16	0.17	0.61	0.23	0.97	0.03	0.00
**A female general friend flushes**															
Fun 1	0.32	0.66	0.02	0.43	0.41	0.17	0.09	0.89	0.02	0.85	0.15	0.00	0.96	0.02	0.02
Fun 2	0.31	0.66	0.04	0.47	0.37	0.16	0.12	0.86	0.03	0.87	0.12	0.01	0.99	0.01	0.00
Fun 3	0.08	0.85	0.08	0.39	0.35	0.26	0.06	0.91	0.03	0.77	0.22	0.01	1.00	0.00	0.00
Risky 1	0.13	0.79	0.08	0.26	0.40	0.33	0.03	0.91	0.06	0.63	0.35	0.02	0.98	0.02	0.00
Risky 2	0.13	0.83	0.04	0.34	0.42	0.24	0.02	0.94	0.04	0.73	0.25	0.02	0.99	0.01	0.00
Risky 3	0.06	0.83	0.11	0.20	0.23	0.57	0.05	0.88	0.07	0.51	0.42	0.08	0.99	0.01	0.00
N		348			808			296			842			267	
%		13.6			31.6			11.6			32.9			10.4	

Note: Response 1 = I would encourage the flusher to reduce or stop drinking, 2 = I would not encourage the flusher to reduce or stop drinking even though I feel that I should, and 3 = I would not do anything because there is no need to. The highest probabilities of responses are shaded in gray.
